# Dimensional asymmetries in social attention: Dynamic head movements elicit opposite attentional biases in vertical versus horizontal dimensions

**DOI:** 10.3758/s13414-026-03351-3

**Published:** 2026-10-13

**Authors:** Felix J. Götz, Paul M. Heineck, Anne Böckler, Andreas B. Eder

**Affiliations:** 1 Institute of Psychology, University of Regensburg, Universitätsstr. 31, 93053 Regensburg, Germany; 2 Institute of Psychology, Julius-Maximilians-University of Würzburg, Würzburg, Germany

**Keywords:** Social attention, Gaze-head integration, Overshoot effect, Spatial cueing, Dimensional asymmetry, Dynamic head movements, Anticipatory inference, Eye-head coordination, Joint attention

## Abstract

**Abstract:**

When observing a person with direct gaze but a horizontally averted head posture, attention often shifts opposite to head direction (overshoot effect). We investigated whether this effect generalizes to vertical head movements and whether it depends on gaze direction information. Across four experiments, centrally presented faces shifted dynamically to averted head positions before a target appeared at a location congruent or incongruent with head direction. Vertical head movements (Experiments 1 and 2) produced head-congruency effects, which were stronger when the eyes were closed. In contrast, horizontal head movements (Experiments 3 and 4) produced classic overshoot effects, which surprisingly were amplified when the eyes were closed. Thus, direct gaze attenuated head-cueing effects but did not alter their direction. Overall, these findings reveal dimensional asymmetries in social attention and demonstrate that gaze-head integration considers spatial context. Implications for hierarchical, holistic, and predictive cue-integration accounts are discussed.

**Open Practices Statement:**

All four experiments were preregistered on the Open Science Framework (OSF; Experiment 1: https://osf.io/qds4x; Experiment 2: https://osf.io/d6rwe; Experiment 3: https://osf.io/xe9a6; Experiment 4: https://osf.io/3pq49). Materials, experimental scripts, stimuli, raw data, and analysis scripts underlying the reported results are publicly available at https://osf.io/aq7zf/ and https://osf.io/j96fx/

**Supplementary Information:**

The online version contains supplementary material available at https://doi.org/10.3758/s13414-026-03351-3.

## Introduction

Determining where another person directs their attention is a fundamental ability in social interaction and nonverbal communication (Emery, 2000; Hessels, 2020; McKay et al., 2021). For instance, when head and gaze are aligned, observers readily establish joint attention and shared situational awareness (Böckler et al., 2011; Frischen et al., 2007). However, the integration of gaze and head cues is not always straightforward. When gaze direction and head orientation are misaligned, systematic biases emerge regarding where another person is perceived to be looking (Moors et al., 2016; Wollaston, 1824). A prominent example is the *overshoot effect* (Langton et al., 2000): When a face with direct gaze is presented together with an averted head posture, observers perceive gaze as shifted slightly in the direction opposite to the head turn, even though gaze is directed at them (Anstis et al., 1969; Balsdon & Clifford, 2018; Gibson & Pick, 1963). Although originally described as a perceptual judgment bias, the effect has also been observed to influence spontaneous attention orienting (e.g., Hietanen, 1999). Moreover, recent work suggests that perceived gaze direction is embedded in broader semantic inferences about a person’s characteristics and states (Landmann et al., 2024). Together, these findings indicate that gaze-head integration is not fixed but flexible and adaptive, reflecting context-sensitive weighting of multiple cues and higher-level inferences about others’ attention and intentions.

The present work addresses an unresolved question: Does the integration of dynamically misaligned gaze-head cues operate according to the same principles in horizontal and vertical space? If so, dynamic misaligned gaze-head configurations should produce analogous patterns of attentional orienting across both dimensions. Conversely, if cue integration depends on spatial dimension, qualitatively different cueing patterns should emerge. Addressing this question first requires understanding how individual social cues influence the orienting of spatial attention, as well as the conditions under which these effects emerge. Social cueing research has consistently shown that centrally presented social signals – such as averted gaze, head orientation, body posture, pointing gestures – shift observers’ attention toward the indicated location, even when they are non-predictive (e.g., Driver et al., 1999; Friesen et al., 2005; Frischen et al., 2007; Langton & Bruce, 1999, 2000). Using variants of Posner’s spatial cueing paradigm (Posner, 1980; Posner & Cohen, 1984; Posner et al., 1980; van der Wel et al., 2018; Zeligman & Zivotofsky, 2018), these studies demonstrate response-time advantages for targets appearing at socially cued locations (Friesen & Kingstone, 1998; for both identification and detection tasks, see Böckler et al., 2011; Hietanen, 2002).^1^ Although these findings are typically interpreted as reflecting relatively automatic reflexive orienting that aligns the observer’s attention with that of another person (i.e., producing joint attention; Richardson & Göbel, 2015), a growing body of evidence demonstrates that these effects are substantially modulated by social context, task demands, beliefs about the observed agent, and other higher-order variables (Dalmaso et al., 2020). This flexibility suggests that attentional orienting reflects flexible integration of available information rather than a rigid response to isolated social cues. Notably, most gaze-cueing paradigms use *dynamic* displays in which a face with direct gaze is replaced by one with averted gaze, thereby implying eye movement (e.g., Richardson & Göbel, 2015), whereas studies investigating misaligned gaze-head configurations – including classic demonstrations of the overshoot effect – have relied predominantly on *static* stimuli without explicit movement information (e.g., Hietanen, 1999, 2002; Langton, 2000; Moors et al., 2015; Pomianowska et al., 2012). Consequently, it remains unclear how gaze and head cues are integrated when dynamic head movement itself signals a shift of attention.

To explain overshooting responses to misaligned gaze-head configurations in the horizontal dimension, several models have proposed variants of a unitary direction-detection mechanism (for an overview, see Table 1). Hierarchical accounts suggest that gaze direction of local eye cues is prioritized over more global head information (Hietanen, 2002; Perrett et al., 1992), whereas holistic accounts propose parallel integration of gaze, head, and body signals to derive an overall attention direction (Langton, 2000; Tanaka & Simonyi, 2016). More recent cue-weighting accounts argue that the relative influence of head and gaze depends flexibly on task demands rather than being fixed (Balsdon & Clifford, 2018).^2^ Despite these differences, all three cue-weighting approaches (hierarchical, holistic, dynamic) largely conceptualize gaze-head integration as a process operating on the *currently* available spatial configuration. An alternative possibility is that observers do not merely integrate currently available cues but also predict how gaze and head are likely to become realigned. A misaligned gaze-head configuration implies that the observed person’s attention has recently shifted or is still changing, allowing observers to anticipate future gaze trajectories. If observed actions are represented in terms of their anticipated goals (Umilta et al., 2001), implied head movement may generate expectations about forthcoming gaze shifts, thereby facilitating joint attention (Atkinson et al., 2018; Richardson & Göbel, 2015; Sebanz et al., 2006). Accordingly, attentional orienting may reflect not only the encoding of the current configuration but also predictive inference about another person’s imminent attentional state. This interpretation is broadly consistent with evidence that dynamic displays induce anticipatory biases associated with implied motion (Hubbard, 2014). From this perspective, overshoot effects may arise because observers anticipate the likely continuation of another person’s attentional shift rather than relying exclusively on the current geometric relationship between gaze and head orientation.

**Table 1:** Theoretical accounts and predictions

Account	Core assumption	Mechanism	*Predictions for the present experiments*
**Hierarchical account** (Perrett et al., 1992; Hietanen, 2002)	Gaze direction dominates head orientation.	Priority is given to eye information over head cues.	With visible gaze, orienting should primarily follow gaze. When gaze direction information is absent (closed eyes), orienting should follow head direction. No dimensional asymmetry predicted.
**Holistic account** (Langton, 2000; Tanaka & Simonyi, 2016)	Gaze and head cues are integrated into a single estimate of attentional direction.	Parallel integration of available social cues.	Orienting reflects the integrated gaze-head configuration. No qualitative difference between vertical and horizontal dimensions predicted.
**Flexible cue-weighting account** (Balsdon & Clifford, 2018)	Cue weights depend on available information and task demands.	Relative weighting of gaze and head cues changes flexibly.	Removing gaze information should increase reliance on head orientation, leading to stronger head-congruent orienting.
**Predictive account** (Moors et al., 2015; Oommen et al., 2004)	Observers anticipate how gaze and head will become realigned.	Dynamic head movement elicits predictions about future attentional states.	Dynamic displays may produce anticipatory orienting. Combined with biomechanical constraints, this account allows different cueing patterns across spatial dimensions.

These accounts were developed largely to explain horizontal overshoot effects and therefore make no explicit predictions about whether the same mechanisms generalize to the vertical dimension. At the same time, they all implicitly assume similar gaze-head integration across spatial dimensions. However, eye-head coordination differs substantially between horizontal and vertical movements because of biomechanical constraints and ecological regularities of eye-head movements. Eye-head coordination research indicates that misalignments occur more frequently along the horizontal than the vertical axis. For example, when looking upward or downward, head and eye movements are often more tightly coupled, whereas people can easily glance left or right while keeping the head relatively stationary. Consequently, the visual system affords greater independence between eye and head movements in the horizontal than in the vertical dimension (Fang et al., 2015; Goossens & Van Opstal, 1997). Ecological accounts likewise emphasize functional asymmetries between upper/lower and left/right space (cf. Previc, 1990). If gaze-head integration is constrained by these biomechanical and ecological regularities, dynamic head movements need not produce identical attentional consequences across spatial dimensions. Instead, anticipated attentional realignment may rely more strongly on head orientation in the vertical dimension, where eye movements alone provide less directional range, but more strongly on gaze information in the horizontal dimension (Götz, 2019; Götz et al., 2020). To amplify the contribution of head information relative to gaze cues, the present research employed dynamic head movements rather than static head postures.

The present study tested these dimensional asymmetries in social attention using dynamic stimuli in which a centrally oriented head shifted to an averted head position. Across four experiments, we manipulated (a) the spatial dimension of head movement (vertical vs. horizontal) and (b) the availability of gaze direction information (open vs. closed). Experiment 1 first asked whether dynamic vertical head movements produce head-congruent orienting or a vertical analogue of the classic overshoot effect. Experiment 2 then tested whether the vertical cueing effect observed in Experiment 1 depended on attention to the vertically displaced eye position rather than head orientation by repeating the paradigm with closed eyes. Experiments 3 and 4 addressed the corresponding questions in the horizontal dimension, first replicating the dynamic overshoot paradigm with direct gaze and then examining whether removing gaze information alters the resulting attentional bias. Based on both flexible cue-weighting (Balsdon & Clifford, 2018) and predictive integration accounts (Moors et al., 2015; Oommen et al., 2004), we expected dynamic head movements to increase reliance on head orientation. In the vertical dimension, this was predicted to produce head-congruent orienting (cf. head-congruency effects in static settings; e.g., Götz, 2019), particularly when gaze direction information was unavailable (Experiment 2). In the horizontal dimension, we expected to replicate the classic overshoot effect when gaze information was available. If this horizontal overshoot primarily reflects competition between gaze and head cues, removing gaze direction information should increase reliance on head orientation and consequently attenuate or even reverse the overshoot effect. Together, these experiments provide a systematic test of whether dimensional asymmetries in attentional orienting reflect differences in how gaze and head cues are integrated across spatial dimensions.

## General methods

### Stimuli and stimulus variants

**Fig. 1:**
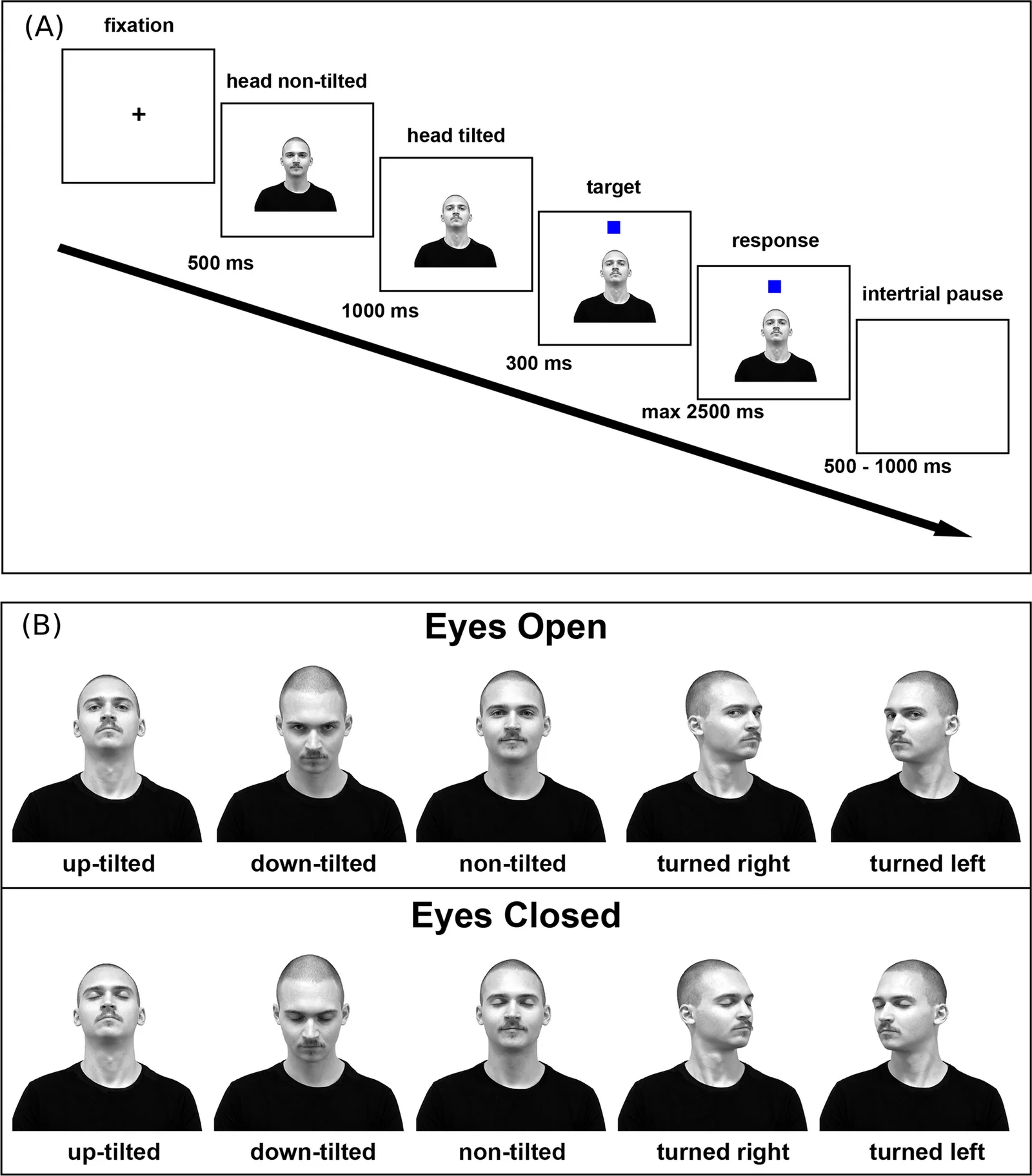
(**A**) Sequence of events in a head-congruent trial of Experiment 1 and (**B**) example pictures of head orientations with open and closed eyes. In the eyes-open stimuli, gaze is always directed at the observer irrespective of head orientation

The stimulus set consisted of 20 portrait-like photographs of four models (two women, two men; five pictures per model), shown frontally and wearing a uniform black shirt (see Fig. 1b). Images were postprocessed to a white background with removed shadows; saturation was reduced (grayscale), and contrast and brightness were matched (Adobe Photoshop CS6). Models were 23–31 years old. For each model, photographs depicted (a) a neutral head orientation and (b) two shifted orientations on the relevant axis. Horizontal variants showed the head turned left or right; vertical variants showed the head tilted up or down. Eye information was manipulated between experiments by using the same head-orientation variants, either with eyes open and gaze directed at the observer (Experiments 1 and 3) or with eyes closed (Experiments 2 and 4). To quantify head turns/tilts, we measured the displacement of the nose tip between the neutral (non-turned/non-tilted) picture and the turned/tilted picture (cf. Götz et al., 2020; Langton et al., 2004). Displacements were: left turns 1.63–2.32 cm (*M* = 1.98, *SD* = 0.25), right turns 1.91–2.42 cm (*M* = 2.08, *SD* = 0.21), down tilts 1.11–1.86 cm (*M* = 1.60, *SD* = 0.30), up tilts 0.84–1.74 cm (*M* = 1.20, *SD* = 0.36). All images were framed such that the midpoint of the image, rather than the head or the eye region, coincided with the center of the screen. This convention kept the shoulders and the lower image border in a constant position across head-orientation variants. Centering on the head would have displaced the lower portion of the image between conditions. Because the depicted person extended further below than above the image midpoint, the head and eye region were located above the screen center. The resulting cue-target distances and a control analysis including target position as a factor are reported in the Online Supplementary Material .

### Trial procedure and task

The experiment was programmed with Inquisit 5 (2016) and was run on computers (Windows 10, 64 Bit) equipped with Corsair STRAFE RGB mechanical keyboards and 24-in. lcd-screens (1,920 × 1,080 px, Benq). All experiments were conducted using QWERTZ keyboards. To minimize potential stimulus-response compatibility (Simon) effects (Simon & Rudell, 1967), vertical head movements (Experiments 1 and 2) were paired with horizontally aligned response keys (F and J), whereas horizontal head movements (Experiments 3 and 4) were paired with vertically aligned response keys (B and Z).

Each trial began with a fixation cross (500 ms), followed by a neutral head image (1,000 ms). The neutral image was then replaced by a shifted head image (left/right turn or up/down tilt). After 300 ms, a blue target (circle or square; 2.45% × 4.00% of screen size) appeared at one of two fixed eccentric locations on the task-relevant axis (see Fig. 1a). Face stimuli were presented centrally. In the horizontal-turn condition, targets were presented to the left or right of the face at positions defined relative to the screen (10% of screen width from the corresponding screen edge), yielding a combined face-target display spanning ~20°of visual angle at a viewing distance of 60 cm. In the vertical-tilt condition, targets were presented above or below the face (10% of screen height from the corresponding screen edge), yielding a combined face-target display spanning ~11° of visual angle. Stimulus and target remained visible until response or 2,500 ms. Incorrect responses triggered an error message (2,000 ms); omissions triggered a speed reminder (2,000 ms). Inter-trial intervals varied between 500 and 1,000 ms.

### Design

Within each experiment, the design fully crossed target identity (circle/square), target location (two positions), and head-shift direction (two directions). Trials were classified as head-congruent when target location matched head-shift direction (left/left, right/right; up/up, down/down), and head-incongruent otherwise. Gaze visibility (eyes open vs. eyes closed) and dimension (horizontal vs. vertical) were manipulated between experiments. Participants completed one practice block (eight trials) and three experimental blocks (72 trials each), with all factor combinations presented equally often per block. Response-key mapping for shape classification was counterbalanced across participants in all four experiments.

### Data processing and statistical analysis

Analyses followed preregistration. Mean response times (RTs) and error rates were computed separately for head-congruent and head-incongruent trials. Incorrect trials were excluded from RT analyses. Participants were excluded when their overall error rate exceeded 3 *SD*s above the sample mean error rate (experiment-specific). RT outliers were removed using individual Tukey thresholds (Tukey, 1977). Head-congruent versus head-incongruent means were compared using two-tailed paired-samples *t*-tests, with effect sizes reported as *d*_z_.

Because reliable a priori estimates of the expected effect sizes were unavailable, we preregistered a minimum sample size of *n* = 38 participants per experiment. A sensitivity analysis indicated that this sample size provides 80% power to detect medium-sized effects of dz ≥ 0.47 in two-tailed paired-samples *t*-tests with α =.05 (G*Power 3.1; Faul et al., 2007).

## Experiment 1

Experiment 1 tested whether dynamic vertical head tilts (up vs. down) with continuously direct gaze would produce vertical head-congruency effects or a vertical analogue of the overshoot effect. To our knowledge, previous studies examining vertical gaze-head configurations did not use direct-gaze stimuli comparable to those employed in horizontal overshoot paradigms (Langton, 2000; Langton & Bruce, 1999). Direction-detection accounts yield opposing predictions. Hierarchical models prioritizing eye-region information (Hietanen, 2002; Perrett et al., 1992) predict a vertical overshoot effect, whereas holistic models assuming parallel integration of gaze and head cues (Langton, 2000; Tanaka & Simonyi, 2016) predict head-congruency effects. Given anatomical and functional asymmetries in vertical versus horizontal eye-head coordination (Fang et al., 2015), observers may rely more strongly on head orientation when inferring vertical shifts of attention. Accordingly, we hypothesized vertical head-congruency effects. This prediction is consistent with flexible cue-weighting accounts (Balsdon & Clifford, 2018) and prior findings using vertically misaligned stimuli (Götz, 2019; Götz et al., 2020).

### Results and discussion

Forty-three volunteers (27 female; *M*_age_ = 28.0 years, *SD* = 7.14, range: 18–53 years) from the Würzburg area participated for financial compensation. In accordance with our preregistration, mean RTs and error rates were computed for head-congruent (down-tilted head and target below the photograph, up-tilted head and target above the photograph) and head-incongruent (down-tilted head and target above the photograph, up-tilted head and target below the photograph) trials. Trials with incorrect responses (2.9% of all trials) were removed from the RT analyses. Moreover, one participant was excluded due to their high error rate (> 3 *SD*s from mean error rate across all participants). In addition, individual outlier thresholds (Tukey, 1977) removed 4.6% of RTs. The remaining mean RTs and mean error rates of head-congruent versus head-incongruent trials were submitted to two-tailed paired-samples *t*-tests.

**Fig. 2:**
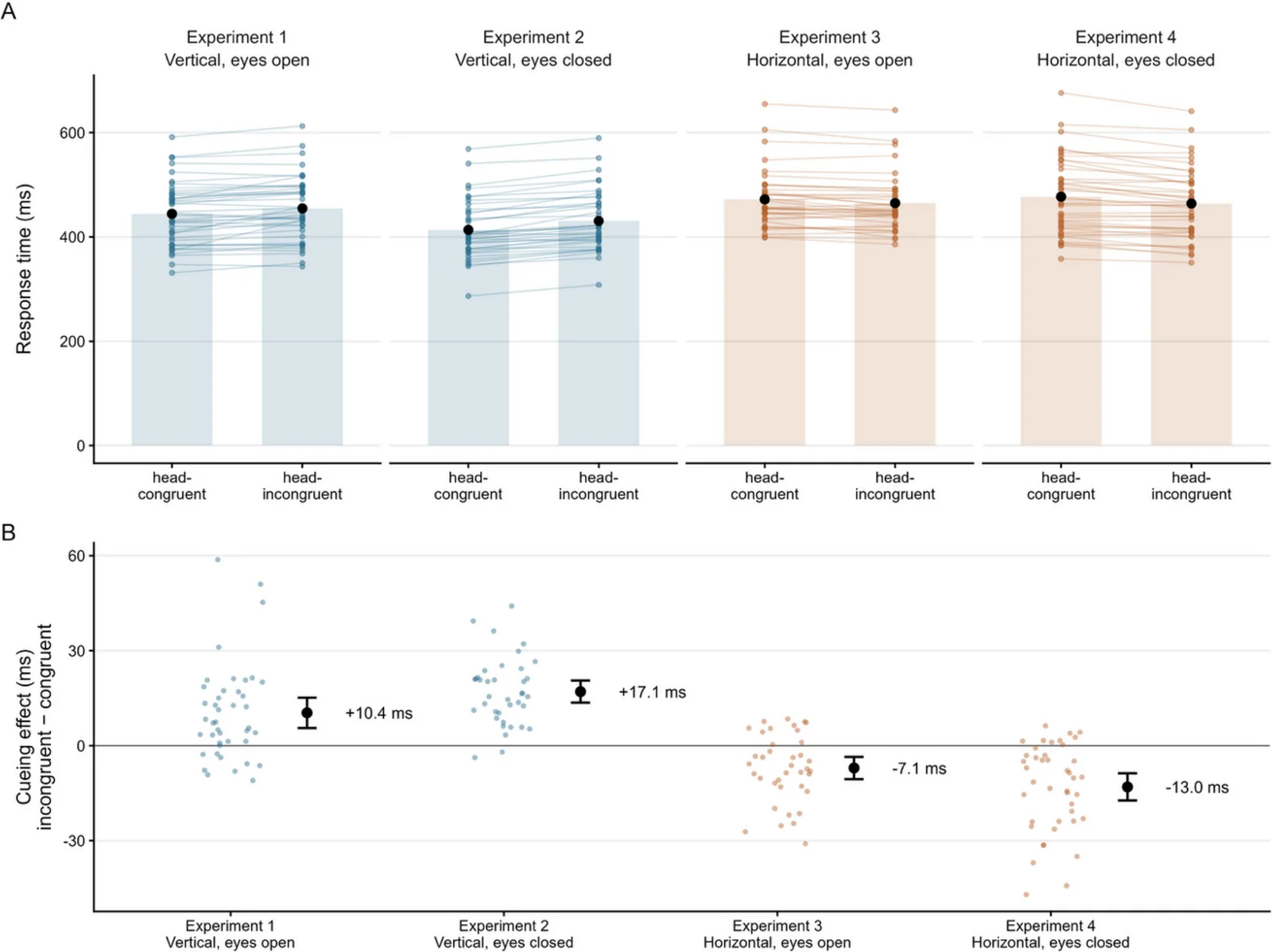
Individual and mean response times as (**A**) a function of congruency of head turn and target location and (**B**) individual cueing effects in Experiments 1–4. Panel A shows individual participants’ mean response times for head-congruent and head-incongruent trials; thin lines connect the two conditions within each participant, and black lines and points indicate condition means. Panel B shows the cueing effect (head-incongruent minus head-congruent) for each participant; black points and error bars indicate the mean and its 95% confidence interval. Positive values indicate attention following head direction, negative values a shift opposite to head direction. In Experiments 1 and 2, head orientation shifted up versus down; in Experiments 3 and 4, it shifted left versus right

In line with our hypothesis, the test showed a significant effect of congruency, *t*(41) = 4.33, *p* <.001, *d*_*z*_ = 0.67. Targets were identified faster if head orientation and target location were congruent (*M* = 443 ms ± *SD* = 61 ms) than if they did not (*M* = 455 ms ± *SD =* 61 ms; see Fig. 2). The analysis of error rates yielded no significant effect of congruency, *t*(41) =−0.40,* p* =.689, *d*_*z*_ = 0.06, which might be due to the low mean error rate of 1.32%.

The vertical head-congruency effect is consistent with a flexible integration of gaze and head cues, as predicted by both the dynamic cue-weighting (Balsdon & Clifford, 2018) and predictive accounts (e.g., Oommen et al., 2004), but difficult to reconcile with more rigid cue-weighting accounts such as the hierarchical account (Perrett et al., 1992). However, although Experiment 1 demonstrated head-congruent orienting, it did not distinguish between two possible explanations. The observed effect could either reflect genuine head-based cueing or simply result from the vertically displaced eye region, which reduced the distance between the direct-gaze signal and the target location (cf. the direct gaze effect; Senju & Johnson, 2009; see also Götz, 2019). Experiment 2 was designed specifically to distinguish between these alternatives by removing directional eye information while leaving head orientation unchanged.

## Experiment 2

Experiment 2 primarily served as a control experiment designed to rule out the alternative explanation that the vertical cueing effect observed in Experiment 1 resulted from the vertically displaced eye position rather than from head orientation itself. More precisely, by presenting identical head movements with closed eyes, it tested whether the vertical cueing effect observed in Experiment 1 depended on subtle eye-position information or instead reflected head orientation itself. Because no directional eye information was available, all current cue-integration accounts predict head-congruent orienting. Thus, the critical question was not which account best predicted the outcome, but whether the effect observed in Experiment 1 would persist after ruling out this alternative explanation.

### Results and discussion

In accordance with our preregistered expected sample size bounds, 39 volunteers (28 female; *M*_*age*_ = 26.4 years, *SD* = 7.64, range: 19–51 years) from the Würzburg area participated in the experiment. Mean RTs and error rates were computed for head-congruent and head-incongruent trials. Trials with incorrect responses (5.7% of all trials) were removed from the RT analyses. One participant was excluded due to the high error rate (> 3 *SD*s from mean error rate). In addition, individual Tukey thresholds (Tukey, 1977) removed 3.9% of trials. The remaining mean RTs and mean error rates of head-congruent versus head-incongruent trials were submitted to two-tailed paired-samples *t*-tests.

There was a significant effect of congruency, *t*(37) =−9.86*, p* <.001, *d*_*z*_ = 1.60. Targets were identified faster when head orientation and target location were congruent (*M* = 414 ms ± *SD* = 57 ms) than when they were not (*M* = 431 ms ± *SD =* 58 ms; see Fig. 2). The analysis of error rates yielded no significant effect of congruency, *t*(37) =−1.60,* p* =.119, *d*_*z*_ = 0.26.

The head-congruency effect replicated the results of Experiment 1. Because the stimuli in the present experiment showed closed eyes, explanations based on direct gaze (Senju & Johnson, 2009) or subtle vertical eye-position cues are unlikely. Together, Experiments 1 and 2 indicate that dynamic vertical head tilts produce robust head-congruent orienting and that this effect persists when gaze information is removed.

We next turned to the horizontal dimension. Experiments 3 and 4 tested whether the overshoot pattern reported for horizontally averted heads generalizes to dynamic displays and whether its direction depends on the availability of gaze information.

## Experiment 3

Experiment 3 examined whether dynamic head turns (left vs. right) with direct gaze reproduce the overshoot pattern typically reported for static misaligned head-gaze configurations. This experiment therefore served as the horizontal analogue of Experiment 1 and provided the baseline for assessing potential gaze-related modulation in Experiment 4.

### Results and discussion

Thirty-eight volunteers (32 female; *M*_*age*_ = 27.3 years, *SD* = 9.34, range: 19–61 years) from the Würzburg area participated in the experiment for financial compensation. Trials with incorrect responses (4.7% of all trials) were removed from the RT analyses. In addition, individual Tukey thresholds (Tukey, 1977) removed 5.0% of trials. Mean RTs and error rates of head-congruent versus head-incongruent trials were submitted to a two-sided paired-samples *t*-test.

The test on RTs showed a significant overshoot effect, *t*(37) = 4.02*, p* <.001, *d*_*z*_ = 0.65. Targets were identified faster if head orientation and target location were incongruent (*M* = 465 ms ± *SD* = 54 ms) than if they were congruent (*M* = 472 ms ± *SD =* 56 ms; see Fig. 2). The error rates analysis yielded no significant effect, *t*(37) =1.68,* p* =.101, *d*_*z*_ = 0.28, which might be due to the low mean error rate of 2.28%.

The RT results replicate the horizontal overshoot effect (Hietanen, 1999) with dynamic stimuli, suggesting that gaze information in the eye region outweighed head orientation when determining attentional direction. Among the cue-weighting accounts, this pattern is particularly consistent with hierarchical accounts of gaze-head integration (Hietanen, 2002; Perrett et al., 1992) and difficult to reconcile with holistic integration models (Langton, 2000; Tanaka & Simonyi, 2016). If horizontal overshoot reflects competition between gaze and head cues, removing directional gaze information should increase reliance on head orientation and produce head-congruency effects. Experiment 4 tested this prediction by repeating the horizontal head-turn paradigm with closed eyes.

## Experiment 4

Experiment 4 tested whether passive viewing of heads turning left or right from a central position, with the eyes closed, would produce faster responses to targets appearing at head-congruent compared to head-incongruent locations. As in Experiment 2, directional gaze information was absent in the stimuli. Even the hierarchical account of Perrett et al. (1992) proposes that head orientation may serve as a cue to another person’s direction of attention when eye information is unavailable (e.g., due to distance or occlusion). Accordingly, we expected the horizontal overshoot effect observed in Experiment 3 to reverse into a head-congruency effect.

### Results and discussion

Forty-two volunteers (28 female; *M*_*age*_ = 30.1 years, *SD* = 11.79, range: 19–71 years) from the Würzburg area participated in the experiment. Trials with incorrect responses (3.4% of all trials) and RT outliers (5.1% of trials) identified according to Tukey (1977) were removed from the RT analysis.

Contrary to our hypothesis, the analysis showed a significant overshoot-like effect, *t*(41) =5.99*, p* <.001, *d*_*z*_ = 0.92. Targets were identified faster when head orientation and target location were incongruent (*M* = 463 ms ± *SD* = 67 ms) than when they were congruent (*M* = 476 ms ± *SD =* 73 ms; see Fig. 2). The error rates analysis yielded no significant effect, *t*(41) =0.41, *p* =.684, *d*_*z*_ = 0.06. Thus, the RT results indicate that in the horizontal dimension, attention was biased opposite to head direction even in the absence of visible gaze direction or eye contact information.

### Explorative comparisons of Experiments 1–4

The following analyses were exploratory and not preregistered and should therefore be interpreted as hypothesis-generating. They were motivated by the descriptive observation that cueing effects appeared numerically larger in the eyes-closed than in the eyes-open experiments. One possible explanation is that direct gaze in the eyes-open conditions introduced an additional attentional signal that attenuated head-based cueing effects, independent of spatial axis (cf. the eye-contact effect; Böckler et al., 2014; Senju & Johnson, 2009). To test this possibility, we examined whether the magnitude of the congruency effect varied as a function of gaze availability within each spatial axis. Because the experiments differed in overall response speed, and the size of a congruency effect can scale with mean RT, each interaction was additionally verified with a linear mixed model fitted to log-transformed single-trial RTs. The models included Congruency, Gaze information, and their interaction as deviation-coded fixed effects, together with by-participant random intercepts and by-participant random slopes for Congruency; degrees of freedom were approximated using Satterthwaite’s method. Sensitivity analyses indicated that the samples were large enough to offer a power of 80% for a minimum effect size of η_*p*_^2^ =.025 for the comparison of Experiments 1 and 2 as well as for the comparison of Experiments 3 and 4.

To assess whether gaze information modulated vertical cueing, RTs from Experiment 1 (eyes open) and Experiment 2 (eyes closed) were pooled and submitted to a 2 (Congruency: head-congruent vs. head-incongruent; within-subjects) × 2 (Gaze information: eyes open vs. eyes closed; between-subjects) mixed ANOVA. The analysis showed a main effect of Congruency, *F*(1,79) = 83.62, *p* <.001, η_*p*_^2^ =.51, indicating faster responses on head-congruent than head-incongruent trials (head-congruency effect). The main effect of Gaze information was also significant, *F*(1,79) = 4.25, *p* =.043, η_*p*_^2^ =.05. Importantly, the Congruency × Gaze information interaction was significant, *F*(1,79) = 5.56, *p* =.021, η_*p*_^2^ =.07, indicating that the vertical head-congruency effect was larger with eyes closed (Experiment 2: head-congruent *M* = 414 ms, head-incongruent *M* = 431 ms) than with eyes open (Experiment 1: head-congruent *M* = 444 ms, head-incongruent *M* = 454 ms). The interaction was confirmed by the linear mixed model on log-transformed single-trial RTs, *F*(1, 79.92) = 6.64, *p* =.012, (*b* = 0.019, *SE* = 0.007, *t* = 2.58). Expressed as a proportion of baseline speed, the vertical head-congruency effect amounted to 2.5% with eyes open and 4.4% with eyes closed, indicating that the larger vertical effect with eyes closed did not merely reflect the overall difference in response speed between the two experiments (standardized within-subject effect size *d*_*z*_ = 1.60 with eyes closed vs. *d*_*z*_ = 0.66 with eyes open).

An analogous post hoc mixed ANOVA compared the horizontal experiments, pooling RTs from Experiment 3 (eyes open) and Experiment 4 (eyes closed). The analysis yielded a main effect of Congruency, *F*(1,77) = 50.62, *p* <.001, η_*p*_^2^ = .40, reflecting overall faster responses on head-incongruent than head-congruent trials (overshoot-like pattern) thus mirroring the same attentional pattern observed in Experiment 3. Critically, the Congruency × Gaze information interaction was significant, *F*(1,77) = 4.30, *p* =.041, η_*p*_^2^ = .05, indicating that the head-opposite attentional bias was a bit larger with eyes closed (Experiment 4: head-congruent *M* = 476 ms, head-incongruent *M* = 463 ms) than with eyes open (Experiment 3: head-congruent *M* = 472 ms, head-incongruent *M* = 465 ms). The main effect of Gaze information was not significant, *F* < 1. As for the vertical axis, this interaction was confirmed by the corresponding linear mixed model on log-transformed single-trial RTs, although the evidence was appreciably weaker, *F*(1, 76.81) = 4.00, *p* =.049, (*b* = −0.011, *SE* = 0.006, *t* = −2.00). In relative terms, the head-opposite bias amounted to 1.6% with eyes open and 2.7% with eyes closed (*d*_*z*_ = −0.65 vs. −0.94). The horizontal modulation should therefore be regarded as tentative.

To summarize, these post hoc comparisons suggest that removing gaze information (eyes closed) was associated with a stronger vertical head-congruency effect, an interaction that remained robust when the overall difference in response speed was controlled by modelling log-transformed RTs at the level of single trials. For the horizontal axis, the corresponding difference was reliable in raw RTs but remained significant in the same trial-level analysis, albeit small and should therefore be interpreted with caution. Because these analyses were exploratory and relied on between-experiment comparisons, the findings should be considered preliminary and motivate preregistered follow-up studies examining gaze availability as a moderator within a single controlled design.

## General discussion

The present research examined how horizontal and vertical head movements influence attentional orienting when gaze and head orientation are misaligned, under conditions of direct gaze and closed eyes using dynamic instead of static stimuli. Across four experiments, a clear dimensional dissociation emerged: In the vertical dimension, attention followed head direction (i.e., head-congruency effects), whereas in the horizontal dimension attention shifted opposite to head turns, replicating the classic overshoot effect with direct gaze (Experiment 3) (Hietanen, 1999; Langton et al., 2000) and revealing a similar attentional pattern even when gaze direction information was absent (Experiment 4). Importantly, these patterns occurred both with direct gaze and when gaze direction information was absent (closed eyes). This pattern directly bears on the question whether observers interpret misaligned gaze-head configurations merely as geometric cues (hierarchical and holistic accounts; Hietanen, 2002; Langton, 2000; Perrett et al., 1992; Tanaka & Simonyi, 2016) or as signals that support predictions about how another person’s attention will be realigned (Moors et al., 2015; Oommen et al., 2004; Pomianowska et al., 2012). The present results favor the latter interpretation, but with an important qualification discussed below.

The dissociation between head-following in the vertical dimension and overshoot in the horizontal dimension suggests that mechanisms integrating gaze-head information may be sensitive to biomechanical and ecological properties of eye-head coordination. Horizontal and vertical eye movements differ in range and coordination patterns (Fang et al., 2015; Tweed et al., 1995), and vertical eye mobility is comparatively constrained (e.g., Yang & Kapoula, 2003). In this context, head orientation may constitute a more reliable predictor of imminent attentional shifts in the vertical dimension, resulting in head-congruency effects. In contrast, the greater independence of horizontal eye movements may foster anticipatory shifts opposite to head direction, consistent with classic overshoot findings (Anstis et al., 1969; Gibson & Pick, 1963; Hietanen, 1999). This vertical-horizontal dissociation is difficult to reconcile with strictly hierarchical accounts, which prioritize gaze direction (Hietanen, 2002; Perrett et al., 1992), or holistic accounts assuming parallel integration of gaze and head cues (Langton, 2000; Tanaka & Simonyi, 2016). Neither framework predicts asymmetries of directional orienting across spatial dimensions, nor do they accommodate robust horizontal head-opposite attentional biases when gaze direction information is absent. Instead, the present findings are more consistent with a predictive interpretation of gaze-head integration as outlined in the *Introduction* (e.g., Moors et al., 2015; Oommen et al., 2004). A dynamically averted head posture implies a shift of attention and invites inference about how gaze and head will be realigned (Moors et al., 2015; Oommen et al., 2004; Pomianowska et al., 2012). From this perspective, attentional orienting reflects not only encoding of the current spatial configuration but also anticipatory inference about the observed person’s imminent attentional state. Although the present experiments do not directly measure predictive expectations, the observed pattern of dimensional asymmetries and gaze-independent effects is mostly consistent with an anticipatory account. This interpretation aligns with goal-based accounts of action representation (Umilta et al., 2001) and joint-attention frameworks (Göbel et al., 2015; Richardson & Göbel, 2015; Sebanz et al., 2006), subject to the following qualification.

Contrary to our expectations, removing gaze direction information by presenting faces with closed eyes did not increase reliance on head orientation and attenuate or reverse the overshoot effect, as predicted by cue-weighting (e.g., Balsdon & Clifford, 2018) and predictive accounts (e.g., Moors et al., 2015; Oommen et al., 2004). Instead, Experiment 4 revealed what appeared to be an even stronger attentional bias opposite to head direction despite the absence of gaze direction information or direct eye contact. Because the classic overshoot effect is typically defined as a consequence of misaligned gaze and head orientation, this result suggests that a similar attentional pattern can emerge even without visible gaze cues, thereby challenging traditional accounts of the overshoot effect. This finding also contrasts with previous studies reporting head-congruent cueing from head orientation (e.g., Hietanen, 1999, 2002; Langton & Bruce, 1999). One important difference is that those studies predominantly employed static head postures, whereas the present paradigm used dynamic head movements. Although dynamic motion might be expected to strengthen reliance on head orientation, the present findings suggest that it simultaneously provides information about the temporal unfolding of another person’s attentional shift. This additional information may engage predictive processes that are largely absent in static paradigms and, in the horizontal dimension, seem to bias attention opposite to head direction. One possibility is that removing gaze information increases ambiguity about another person’s current attentional state, encouraging observers to rely more strongly on inferences about the meaning of the observed movement. For example, if the head movement is interpreted as an avoidance or disengagement signal rather than as a simple directional cue, observers may infer that the observed person is redirecting attention away from a location and consequently orient toward the likely source of that attentional shift. These interpretations are consistent with accounts proposing that social perception incorporates higher-level inferences about others’ intentions and action structure (Balsdon & Clifford, 2018; Teufel et al., 2009, 2010) as well as with predictive perspectives on gaze-head processing (e.g., Moors et al., 2015; Oommen et al., 2004). However, the present data do not allow us to determine whether these effects reflect primarily inference-based (top-down) processes or more reflexive (bottom-up) mechanisms.

A complementary perspective concerns how observers infer the intentional relation between the observed person and the environment. Research on gaze perception often distinguishes between dyadic interpretations, where attention is directed toward the observer, and triadic interpretations, where attention is directed toward objects in the surrounding world (Barresi & Moore, 1996; Senju & Johnson, 2009). In the present paradigm, head movements may have been interpreted as signals that the observed person’s attention was being reallocated toward – or away from – locations in the environment, thereby prompting anticipatory orienting toward – or away from – potential targets. From this perspective, the results are consistent with accounts proposing that observers engage in “attention reading,” dynamically integrating head orientation, gaze cues, and motion information to infer where another person’s attention will be directed next (Teufel et al., 2009, 2010; see also Tufft & Richardson, 2026).

The present findings should be interpreted in light of several limitations. First, RTs provide only an indirect measure of attentional orienting compared to eye tracking. Second, the stimulus set consisted of a limited number of portrait-like photographs from four models. Although restricting stimulus variability may reduce perceptual noise (Götz, 2019; Götz et al., 2020), future studies should test whether the observed dimensional dissociation generalizes across a broader range of identities and head morphologies. Third, horizontal and vertical manipulations were implemented in separate experiments. Although a within-subject comparison would allow a more direct statistical evaluation of dimensional asymmetries, it could also introduce carry-over effects by exposing participants to multiple spatial dimensions or gaze conditions within the same experiment. Such exposure may encourage explicit comparisons between conditions or strategic adaptations that could influence subsequent cue processing. We therefore adopted a between-participant design as the more conservative approach, while acknowledging that future studies should determine whether the present findings replicate within a fully crossed within-participant design. Finally, although the present interpretation emphasizes anticipatory inference, the experiments did not directly measure perceived intentionality or expected gaze realignment. Future work could combine attentional measures with explicit expectation judgments to more directly test predictive accounts (cf. Oommen et al., 2004; Teufel et al., 2010).

The present experiments demonstrate a striking dimensional dissociation in attentional responses to dynamic misaligned gaze-head cues. When a head movement created a misalignment between gaze and head orientation, attention followed head direction in the vertical dimension but shifted opposite to head direction in the horizontal dimension. To our knowledge, this is the first demonstration that horizontal overshoot and vertical head-congruency effects coexist within the same dynamic paradigm. Importantly, both effects emerged under conditions of both direct gaze and closed eyes and were amplified when gaze orientation information was absent. This pattern is difficult to reconcile with strictly hierarchical gaze-prioritization accounts (Hietanen, 2002; Perrett et al., 1992) or holistic cue-integration models (Langton, 2000; Tanaka & Simonyi, 2016). Instead, the results favor a more flexible interpretation of gaze-head integration in which observers infer how another person’s attention will be realigned (Moors et al., 2015; Oommen et al., 2004). Notably, the attentional bias opposite to head direction persisted – and was even amplified – when visible gaze information was absent. This finding suggests that the classic horizontal overshoot effect may not arise exclusively from gaze processing but may additionally reflect inferences about disengagement or avoidance. Beyond this qualification, the findings suggest that gaze-head integration is constrained by biomechanical and ecological properties of eye-head coordination (Fang et al., 2015; Previc, 1990). By demonstrating that horizontal overshoot and vertical head-congruency effects arise from the same misaligned gaze-head configurations under dynamic conditions, the present findings extend classic overshoot research (Anstis et al., 1969; Langton et al., 2000) and highlight the context sensitivity of social attention (Balsdon & Clifford, 2018; Teufel et al., 2009). Taken together, the present findings suggest that direction detection in social perception is best understood as an anticipatory process in which mechanisms supporting attention following integrate gaze cues, head movements, and ecological constraints to infer how another person’s attention is likely to be realigned.

## Supplementary Information

Below is the link to the electronic supplementary material.

Supplementary file1 (DOCX 16 KB)

## Data Availability

Materials, stimuli, and raw data underlying the reported results are publicly available on the Open Science Framework (OSF) at https://osf.io/aq7zf/ and https://osf.io/j96fx/

## Footnotes

1 In the present article, *cueing* denotes attentional orienting effects elicited by social stimuli independently of their spatial predictiveness. The cues were non-informative: target probability was identical at cued and uncued locations. Thus, any cueing effects cannot be attributed to informational validity but instead reflect modulation of attentional processing or cognitive priming (Sudevan & Taylor, 1987).

2 Specifically, Balsdon and Clifford (2018) showed that, when observers judged gaze direction (left vs. right), head orientation repulsed perceived gaze away from the direction of the head turn, producing an overshoot effect. By contrast, when observers judged whether gaze was direct or averted, the same head-gaze configurations produced little or no bias, indicating that identical head-gaze configurations are reweighted as a function of task demands and judgment context.

## Code availability

Experimental scripts and analysis scripts underlying the reported results are publicly available on the OSF at https://osf.io/aq7zf/ and https://osf.io/j96fx/
