# Supplementary material for: Dimensional asymmetries in social attention: Dynamic head movements elicit opposite attentional biases in vertical versus horizontal dimensions
Source: Atten Percept Psychophys. 2026 Oct 13;88(7):205. doi: 10.3758/s13414-026-03351-3 (PMC13661678; doi:10.3758/s13414-026-03351-3)
Supplement: Supplementary file 1 [file 13414_2026_3351_MOESM1_ESM.docx]

**Supplementary Material of the Manuscript:
Dimensional Asymmetries in Social Attention:** **Dynamic Head Movements Elicit Opposite Attentional Biases in Vertical vs. Horizontal Dimensions**

Felix J. Götz^1 2^, Paul M. Heineck^2^, Anne Böckler^2^, & Andreas B. Eder^2^

^1^University of Regensburg, Germany

^2^Julius-Maximilians-University Würzburg, Germany

**Effect of target position in Experiment 1 and 2**

Because face images were framed on the midpoint of the image rather than on the head, upper targets were somewhat closer to the head than lower targets. This asymmetry is orthogonal to the congruency manipulation: within each target position, congruent and incongruent trials share an identical cue-target distance, so the framing can produce a main effect of target position but cannot by itself generate a congruency effect. Adding target position as a within-subjects factor to the analysis confirmed this. The Congruency × Target Position interaction was negligible in Experiment 1, *F*(1,41) = 0.75, *p* = .393, η_G_² < .001, and in Experiment 2, *F*(1,37) = 0.70, *p* = .409, η_G_² < .001, with Bayesian paired-samples t tests indicating moderate evidence against a modulation (Experiment 1: *BF*₀₁ = 4.23; Experiment 2: *BF*₀₁ = 4.14). The cueing effect was reliable at both positions separately (Experiment 1: upper 8.41 ms, *p* = .022, lower 12.42 ms, *p* < .001; Experiment 2: upper 15.70 ms, *p* < .001, lower 18.49 ms, *p* < .001). A main effect of target position emerged in Experiment 1, *F*(1,41) = 11.99, *p* = .001, η_G_² = .005, but not in Experiment 2, *F*(1,37) = 0.53, *p* = .470, η_G_² < .001.
